# Amniotic Membrane Extract Eye Drop Promotes Limbal Stem Cell Proliferation and Corneal Epithelium Healing

**DOI:** 10.22074/cellj.2019.5423

**Published:** 2018-08-07

**Authors:** Niloufar Shayan Asl, Farhad Nejat, Parvaneh Mohammadi, Abdolhossein Nekoukar, Saeed Hesam, Marzieh Ebrahimi, Khosrow Jadidi

**Affiliations:** 1 Department of Stem Cells and Developmental Biology, Cell Science Research Centre, Royan Institute for Stem Cell Biology and Technology, ACECR, Tehran, Iran; 2 Vision Health Research Center, Semnan University of Medical Sciences, Semnan, Iran; 3 Animal Core Facility, Reproductive Biomedicine Research Centre, Royan Institute for Biotechnology, ACECR, Tehran, Iran; 4 Department of Epidemiology and Reproductive Health, Reproductive Epidemiology Research Centre, Royan Institute for Reproductive Biomedicine, ACECR, Tehran, Iran

**Keywords:** Amniotic, Corneal Healing, Proliferation, Stem Cell

## Abstract

**Objective:**

Human amniotic membrane (HAM) is used as a supporter for limbal stem cell (LSC) expansion and corneal surgery. The aim of study is to use HAM extracts from healthy donors to enhance proliferation of LSCs *in vitro* and *in vivo*.

**Materials and Methods:**

In this interventional experimental study, the effective and cytotoxic doses of the amniotic membrane extract eye drops (AMEED) was assessed by adding different concentrations of AMEED (0-2.0 mg/ml) to LSC cultures for 14 days. Subsequently, the expression levels of ATP-binding cassette sub-family G member 2 (ABCG2, a putative stem cell marker), cytokeratin 3 (*K3*, corneal maker), *K12* and K19 (corneal-conjunctival cell makers) were assessed by real-time polymerase chain reaction (PCR). In the second step, the corneal epithelium of 10 rabbits was mechanically removed, and the right eye of each rabbit was treated with 1 mg/ml AMEED [every 2 hours (group 1) or every 6 hours (group 2)]. The left eyes only received an antibiotic. The corneal healing process, conjunctival infection, degree of eyelid oedema, degree of photophobia, and discharge scores were evaluated during daily assessments. Finally, corneal tissues were biopsied for pathologic evidences.

**Results:**

In comparison to the positive control [10% foetal bovine serum (FBS)], 0.1-1 mg/ml AMEED induced LSC proliferation, upregulated ABCG2, and downregulated *K3*. There were no remarkable differences in the expression levels of *K12* and K19 (P>0.05). Interestingly, in the rabbits treated with AMEED, the epithelium healing duration decreased from 4 days in the control group to 3 days in the two AMEED groups, with lower mean degrees of eyelid oedema, chemosis, and infection compared to the control group. No pathologic abnormalities were observed in either of the AMEED groups.

**Conclusion:**

AMEED increases LSCs proliferation *ex vivo* and accelerates corneal epithelium healing *in vivo* without any adverse effects. It could be used as a supplement for LSC expansion in cell therapy.

## Introduction

For the past 75 years, human amniotic membrane (HAM) has been used in ocular surgery and as a supporter for limbal stem cell (LSC) expansion (1). The anti-inflammatory, anti-scarring, anti-microbial, anti-angiogenic, antifibrotic effects, and low immunogenicity of HAM (1, 2) make it suitable for surgical applications without necessitating the use for systemic immunosuppressive drugs. HAMs produce growth factors that can promote re-epithelialization of the cornea, including epidermal growth factor (EGF), keratinocyte growth factor (KGF), vascular endothelial growth factor (VEGF), basic fibroblast growth factor (bFGF), and platelet-derived growth factor (PDGF) (2, 3). The presence of structural proteins such as collagen (I, III, VI, and VII), laminin, fibronectin, lumican, and osteoglycin can help to explain the observed epitheliotrophic effects of HAM (2-4). Thus, the specific structure of HAM leads to the promotion of reepithelialization and other aspects of the corneal healing process. In particular, HAM induces LSC migration, inhibits apoptosis, and maintains epithelial progenitor cells within the LSC niche (5, 6). 

Advances in stem cell research that include the development of laboratory techniques for isolation and maintenance of LSCs and improvements in surgical techniques have opened a new chapter for the application of bioengineered grafts by enabling *ex vivo* LSC expansion. HAM (either intact or denuded) was the first tissue used as a carrier for *ex vivo* LSC expansion (7, 8). However, the potential disadvantages of amniotic membrane transplantation include donor variation (9), increased risk of viral infections due to the use of fresh tissue, difficulties in HAM manipulation, increased surgery time, and increased risk of complications such as granuloma formation, giant papillary conjunctivitis, and patient discomfort (10).

In recent years, several studies have researched the use of homogenates or extracts of amniotic membrane for the treatment of ocular surface disease. These studies showed that the extracts were able to reduce inflammation and cause the epithelium to develop a more regular and compact appearance; further, all of the patients reported an improvement in symptoms at 15-20 days after treatment. Thus, amniotic membrane extracts appear to be effective for the treatment of certain ocular disorders without necessary to surgical skill (11-13).

However, no research has been conducted to evaluate the effect of HAM extracts on LSC proliferation and differentiation *ex vivo*. Questions about whether amniotic membrane extract can be used as a standardized supplement for LSC culture *ex vivo* (as part of cellular therapy) and whether it can be used as an *in vivo* treatment remain unresolved. Thus, the aim of this study is to prepare standardized HAM-derived eye drops and determine whether amniotic membrane extract eye drops (AMEED) can be an effective supplement for LSC expansion *ex vivo* and promote healing of corneal damage in a rabbit model. 

## Materials and Methods

In this interventional experimental study, we used AMEED as a supplement for expansion of LSCs *in vitro* and promote corneal healing in a rabbit model.

### Preparation of amniotic membrane extract

Cryopreserved HAMs were obtained from the Amniotic Membrane Bank of Royan Institute, Tehran, Iran, that had Ethical approval for HAMs banking (EC/92/10/72). Healthy donors selected based on medical history questionnaires eached signed an informed consent for study participation. All HAMs were negative for human immunodeficiency virus (HIV I and II), hepatitis B virus (HBV), hepatitis C virus (HCV), human T-cell lymphotropic virus (HTLV I and II), cytomegalovirus (CMV), and bacterial infections. 

The HAMs were washed with Mg^2+^-and Ca^2+^-free phosphate-buffered saline (PBS, pH=7.2, L182-01, BioScience, UK) that contained 1000 U/ml penicillin and 50 µg/ml streptomycin (pen/strep, 15070-063, Gibco Life Technologies, USA). Next, the HAMs were cut into small pieces and submerged in liquid nitrogen. The frozen tissues were manually ground into a fine powder, weighed, mixed with distilled water at a 1:1 (w/v) ratio, and homogenized by a sonicator (UP200S, Hielscher Ultrasonics GmbH, Teltow, Germany) on a 20% duty cycle for 10 minutes. The homogenate was centrifuged at 4000 g at 4°C for 10 minutes and at 15000 g at 4°C for 5 minutes to remove the cell debris. The supernatant was collected and filtered using a 0.2-µm filter, and we measured the total protein content as explained below. The final product, AMEED, was aliquoted at a concentration of 1 mg/ml to a final volume of 5 mL and stored at -70°C. For long-term storage, several samples were lyophilized (Christ Alpha 1-2 LDplus, Germany) to maintain the bioactivity of the proteins (including the growth factors).

### Measurement of total protein and growth factor concentrations

The total protein in each batch of AMEED was assessed using a standard Bradford protein assay. Briefly, 20 µl of each sample and a diluted standard that contained 10 µg/ µl .-globulin were added to the wells of a 96-well plate (in duplicate), followed by the addition of 500 µl Bradford buffer (5000006, Bio-Rad Laboratories, Inc., Hercules, CA, USA) to each well and mixed. The optical density at 595 nm was then measured using a spectrophotometer (Multiskan Spectrum, Thermo Fisher Scientific Oy, Vantaa, Finland).

The concentrations of EGF, KGF, hepatocyte growth factor (HGF), and interleukin-1 receptor antagonist (IL1RA), as important amniotic membrane proteins necessary for epithelial regeneration (14), were assessed using commercially available enzyme-linked immunosorbent assay (ELISA) kits (Catalogue no.: DEG00, DKG00, DHG00, and DRA00B, R & D Systems Inc., Minneapolis, MN, USA) according to the manufacturer’s protocols. Four batches of AMEED were used for this growth factor analysis. The stability of the growth factors was tested after one month to one year of storage at -70°C, after 7 days of storage in a refrigerator (2-8°C), and after 2 days of storage at room temperature.

### Limbal stem cell explant culture

We obtained normal human eye globes from the Central EyeBank of the Islamic Republic of Iran following approval by Royan institutional review board (EC90/1039). The LSCs were cultured based on our previously published method (8, 15). Briefly, the fresh limbal region was removed, washed, and treated with Dispase II (1.2 U/ml in Mg^2+^-free and Ca^2+^-free Hanks’ balanced salt solution; 17105-041, Gibco, Auckland, NZ) at 37°C for 5-10 minutes, after which we carefully removed the stromal layer. The tissue was then rinsed with Dulbecco’s Modified Eagle’s Medium Nutrient Mixture F-12 (DMEM/F12, 1760148, Gibco Life Technologies, USA) that contained 10% foetal bovine serum (FBS, Gibco Life Technologies, USA), cut into small cubes of approximately 1-2 mm in length, and cultured on a six-well plate (one cube was used per well). The explants were cultured in limbal medium (LM) comprised of DMEM/F-12 supplemented with 0.5% dimethyl sulfoxide, (DMSO, Sigma, Steinheim, Germany), 2 ng/ml human EGF (Sigma, Germany), 5 µg/ml insulin (Sigma, Germany), 5 µg/ml transferrin (Sigma, Germany), 5 ng/ml selenium (Sigma, Germany), 0.5 µg/ml hydrocortisone (Sigma, Germany), 50 µg/ml gentamicin (Sigma, Germany), and 1.25 µg/ml amphotericin B (Sigma, Germany). AMEED were added to LM at final concentrations of 0.1 0.5, 1.0, and 2.0 mg/ ml. Explants cultured in serum-free LM were used as the negative control and LM supplemented with 10% FBS was the positive control. The cultures were incubated in a humidified incubator in 5% CO_2_ for 14 days, and the medium was replaced every 2 days. The extent of each outgrowth was monitored using an inverted phase- contrast microscope and photographed. The diameter of ImageJ (version 1.50b, National Institute of Health, MD, USA) where the pixel to area conversion was set using the scale bar. To calculate the percentage of outgrowth, the diameter of area covered by LSCs divided on diameter of plate and then multiplied by 100. 

### Limbal stem cell proliferation and cytotoxicity assay

We evaluated the proliferation rate of LSCs by dissociating the cells from 7-day explant cultures. Then, 5×10^3^ single cells were seeded in 96-well plates and cultured in the presence or absence of AMEED for 5 days. The final concentrations of AMEED in LM media were 0.1, 0.5, 1.0, and 2.0 mg/ml. Serum-free LM was the negative control and LM supplemented with 10% FBS was the positive control. At the end of the culture time, the cells were manually counted and we calculated the growth rate by dividing the number of the cells at test group on cell number at negative control.

In order to evaluate the cell cytotoxicity of AMEED, single LSCs were treated for 24 hours in the presence/ absence of AMEED and then subjected to assay with 3-(4,5-dimethylthiazol-2-yl)-2,5-diphenyltetrazolium(MTT, Sigma, Germany) in accordance with the manufacturer’s protocol. 

### Quantitative real-time polymerase chain reaction

Total cellular RNA was extracted from LSCs in the AMEED and control groups at day14 of the explantculture. We carried out cDNA synthesis using a RevertAid H Minus First Strand cDNA Synthesis Kit(Fermentas Life Sciences, USA) in accordance with the manufacturer’s protocol. The cDNA was then amplified using quantitative real-time PCR (qRT- PCR) in the presence of primers specific to the ATP- binding cassette sub-family G member 2 (*ABCG2*) and *P63* (putative stem cell markers), cytokeratin 3 (*K3*, corneal epithelial marker), and *K19* and *K12* (corneal-conjunctival epithelial cell markers) as listed in Table 1. Relative quantification of mRNA using the comparative cycle threshold (C_t_) method was performed with a StepOnePlus Real-Time PCR System (Applied Biosystems, Foster City, CA, USA). The data were analysed by the 2^-ΔΔCt^ method to calculate the fold change in gene expression and normalized to the expression level of an endogenous reference gene, glyceraldehyde 3-phosphate dehydrogenase (*GAPDH*). 

### Rabbit model of corneal defect and treatment

We randomly divided 10 healthy adult male rabbits that weighed 1.5-2.0 kg into two groups. The procedures were performed under general anaesthesia by intramuscular administration of ketamine (35 mg/kg of body weight) and xylazine (5 mg/kg of body weight). Systemic or local immunosuppressive agents were not used in this study. A surgeon used an 8-mm diameter ring to mechanically remove the corneal epithelium from each of the rabbit’s eyes (16). According to our *in vitro* results, 0.1-1 mg/ml of AMEED were effective doses for expansion of LSCs. Therefore, we selected the 1 mg/ml dose of AMEED for animal treatment due to the flow of tears in the eye. A total of 5 rabbits were treated with one drop of AMEED (1 mg/ ml) in their right eyes every 2 hours. The other 5 rabbits were treated with one drop in their right eyes every 6 hours. The antibiotic chloramphenicol was administered every 6 hours in both eyes. The left eyes (control group) only received chloramphenicol to avoid any bacterial infection. All the animals were assessed daily for 6 days by using a slit-lamp microscope to monitor the wound healing process. 

**Table 1:** Oligonucleotide primers used for real-time polymerase chain reaction


Gene name	Gene symbol	Primer sequences (5′-3′)	Product length (bp)

Glyceraldehyde 3-phosphate dehydrogenase	*GAPDH*	F: CTC ATT TCC TGG TAT GAC AAC GA	121
		R: CTT CCT CTT GTG CTC TTG CT	
Protein p63	*P63*	F:TTT CAG AGG CAA TCC ACA CA	137
		R: ATG CAT GCA AAT GAG CTC TG	
ATP-binding cassette sub-family G member 2	*ABCG2*	F:CTC TTC TTC CTG ACG ACC AAC C	515
		R: CAC ACT CTG ACC TGC TGC TAT G	
Cytokeratin 3	*KRT3*	F: AGA CTT CAA GAA GAA ATA TGA G	141
		R: TCA TCT ATC AAG GCA TCC AC	
Cytokeratin 12	*KRT12*	F: TGC GAG CTC TAG AAG AGG CTA	255
		R: CCT CGT GGT TCT TCT TCA TGT A	
Cytokeratin 19	*KRT19*	F: TGA GGT CAT GGC CGA GCA GAA C	216
		R: CAT GAG CCG CTG GTA CTC CTG A	


### Outcome measurements 

Subjective symptoms that included eyelid oedema, chemosis, conjunctival injection, and conjunctival infiltration were assessed using a 0-3 scale for symptoms, as follows: 0 (no), 1 (mild), 2 (moderate), and 3 (severe). Fluorescein staining was used to evaluate the corneal epithelial defect (CED) site. The diameter of each defect was observed by a slit-lamp microscope and measured in millimetres by image analysis software.

### Histopathologic evaluation

We randomly selected rabbits from the AMEED and control groups for histopathologic evaluation of the healing cornea. Briefly, randomly selected rabbits were euthanized by anaesthetic drugs at 2 weeks (n=2), one month (n=2), and 3 months (n=1) after treatment. The rabbit’s eyes were enucleated, fixed in 10% formaldehyde, embedded in paraffin, cut into 5-µm sections, and stained with hematoxylin and eosin (H&E).

### Statistical analysis

Means and standard deviations were calculated based on at least three biological experiments. Statistical analyses of the quantitative variables with normal distribution were carried out using one-way analysis of variance (ANOVA). For multiple comparisons, we used Tukey’s test. A mixed-model analysis was used to compare the variables between time points. P<0.05 was considered to indicate statistical significance. All of the statistical analyses were performed using SPSS 17.0 statistics software (SPSS Inc., Chicago, IL, USA). 

## Results

### Amniotic membrane extract eye drops promotes limbal stem cell growth 

**Fig.1:**
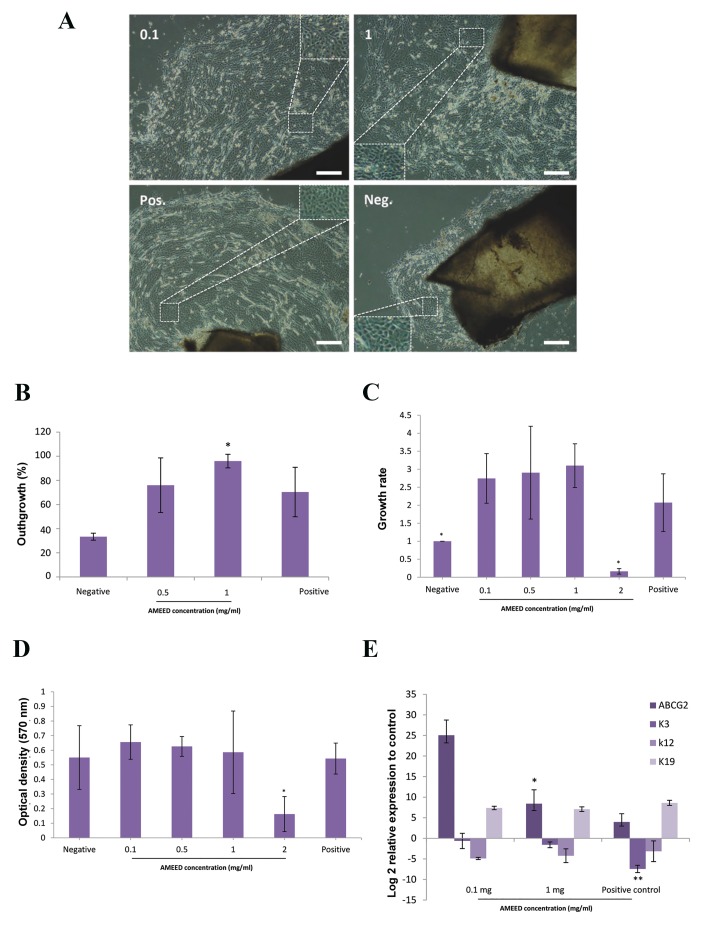
Limbal cell growth with amniotic membrane extract eye drop (AMEED) exposure and gene expression chart. A. Limbal explants grown in limbal medium (LM) supplemented with 0.1 and 1 mg/ml of AMEED as the test groups, 10% foetal bovine serum (FBS) as the positive control, and serum-free LM as the negative control (scale bar: 200 µm), B. The diameter of the area covered by limbal stem cells (LSCs) divided on the diameter of the culture area of the plate, then multiplied by 100, C. LSCs were dissociated from explant cultures, re-seeded, and incubated with AMEED to evaluate the growth rate in each group, D. Cell cytotoxicity assessed 24 hours post-treatment by AMEED on dissociated cells from an explant culture using 3-(4,5-dimethylthiazol-2-yl)-2,5diphenyltetrazolium (MTT), and E. Expression of ATP-binding cassette sub-family G member 2 (ABCG2, stemness related marker), *K3*, *K12* (corneal related marker), and K19 (conjunctival/corneal differentiation marker) were evaluated in 14-day explant cultured cells by quantitative real-time polymerase chain reaction (qRT-PCR). Glyceraldehyde 3-phosphate dehydrogenase (GAPDH) was the internal control and data was normalized by the negative control. Each bar represents the mean ± SD of at least three different experiments. *; P=0.01 and **; P=0.001.

We added different concentrations of AMEED to the LSC culture medium to understand the effect of AMEED on LSC growth. The epithelial sheets grown from the limbal explants in the AMEED and positive control groups had regular margins (Fig .1A). Most cells were small and circular, with a high nucleus-tocytoplasm ratio during the 14-day culture period. In the negative control group, the outgrowth had an irregular margin, and most of the cells were differentiated epithelial cells that had a large cytoplasm and small nucleus. 

The percentage of outgrowth was greater for the cells treated with AMEED at 1 mg/ml (P<0.01, Fig .1B). Compared to the negative control group, we observed a higher rate of epithelial growth for the cells treated with 0.1, 0.5, and 1.0 mg/ml AMEED (P=0.01, Fig .1C). However, compared to the positive control group, there were no differences in cells treated with 0.1, 0.5, and 1.0 mg/ml AMEED (P>0.05, Fig .1C). Interestingly, AMEED at high concentrations (=2 mg/ ml) significantly decreased LSC growth (P<0.001, Fig .1C), which might have been due to cytotoxicity (Fig .1D).

### Amniotic membrane extract eye drops limits corneal differentiation and promotes limbal stem cell expansion

We investigated whether AMEED increased the number of LSCs or differentiated cells by assessing the expression levels of ABCG2, *K3*, *K12*, and K19 using qRT-PCR. All four genes expressed in the cultured cells, but their levels differed in the AMEED and control groups (Fig .1E). Compared to the cells in the negative control group, ABCG2 and K19 upregulated in cells treated with 0.1-1 mg/ml AMEED and those in the positive control group. However, *K3* and *K12* downregulated in the AMEED and positive control groups in comparison to the negative control group. Interestingly, cells treated with 0.1-1 mg/ml AMEED expressed a higher level of ABCG2 mRNA and a lower level of *K3* mRNA compared with the positive control group (P<0.05, Fig .1E). In contrast, there were no differences in the expressions of *K12* and K19 between the AMEED groups and the positive control group (P>0.05, Fig .1E). 

### Amniotic membrane extract eye drops promotes corneal healing in a rabbit model

We evaluated the effect of AMEED on corneal healing. Corneal defects were mechanically produced in 10 rabbits, after which AMEED was administered every 2 hours in the first group and every 6 hours in the second group. 

**Fig.2:**
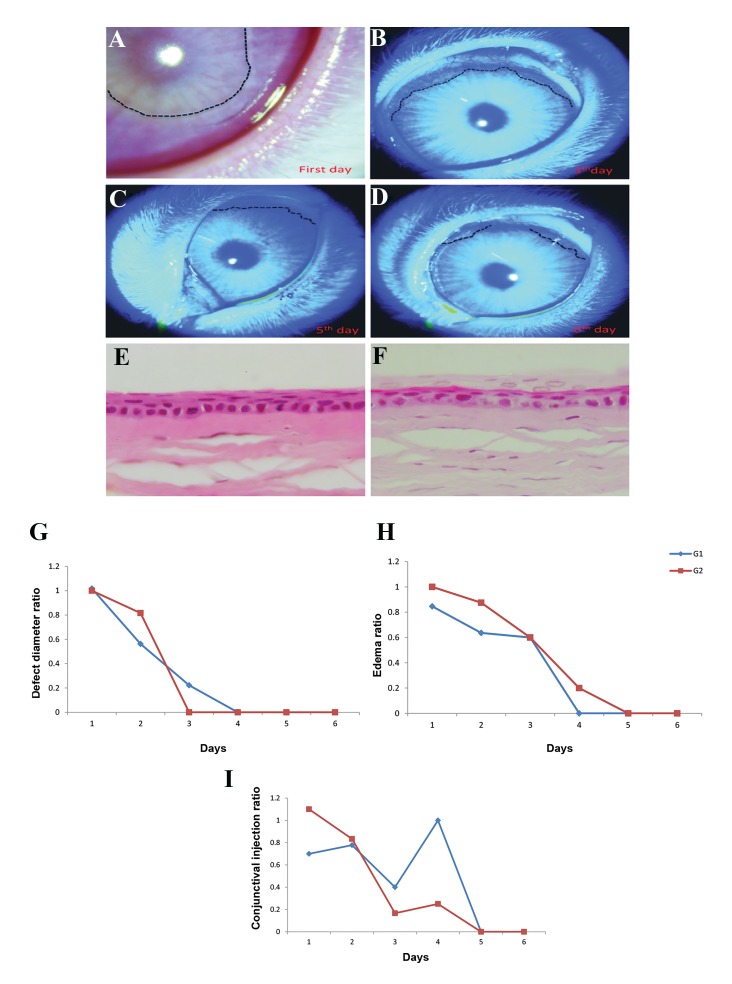
Corneal epithelium healing, hematoxylin and eosin (H&E) staining of cornea and healing analysis in animal models. A-D. Corneal epithelium healing during 6-days post-corneal defect. Group 1 received amniotic membrane extract eye drops (AMEED) each 2 hours. Fluorescein did not stain the intact cornea; rather, the wounded area was stained. Dots show the fluorescein positive area, E. Prominent mitoses at the level of the basal epithelium in both the control and test corneas of a group 1 rabbit 3 months post-treatment, (H&E, ×1000), F. Superficial epithelial cells with pale hypochromatic and plump spindle-shaped nuclei and pale cytoplasm at the cornea section seen in both groups (both left and right eyes) at 3 months post-treatment(H&E, ×1000), G. Diameter of rabbits’corneal epithelium defect (CED), H. Lid edema, and I. Conjunctiva injection in group 1 (G1) and group 2 (G2) in comparison to their controls at 6 days of treatment.

The modelling healed during 6 days in the AMEED group and was delayed in the control group (Fig .2A-D). Histopathologic observations confirmed that mitosis was normal in the healing area at 2 weeks post- treatment in both AMEED groups (Fig .2E, F). 

The diameter of the CED in both AMEED groups was significantly lower than the control group (P=0.017). The healing time was shorter in the second group that received AMEED each 6 hours on a daily basis (Fig .2G). The mean degree of eyelid oedema and chemosis (Table 2) were lower in the AMEED groups. This finding was particularly notable in the first group compared with the control group (P=0.006, Fig .2H, Table 2).

**Table 2:** Chemosis, discharge, and photophobia symptoms after corneal healing


Chemosis												
G1 (2 hours)							G2 (6 hours)					

Days	D1	D2	D3	D4	D6	D5	D1	D2	D3	D4	D5	D6
1 (R)	0	0	0	0	0	0	Mi	0	0	0	0	0
1 (L)	Mo	Mi	0	0	0	0	Mi	Mi	0	0	0	0
2 (R)	0	0	0	0	0	0	Mi	0	0	0	0	0
2 (L)	S	Mo	0	0	0	0	Mi	Mi	0	0	0	0
3 (R)	Mo	Mi	0	0	0	0	Mi	0	0	0	0	0
3 (L)	Mo	Mo	Mi	0	0	0	Mi	Mi	0	0	0	0
4 (R)	Mo	Mo	Mi	0	0	0	S	Mo	0	0	0	0
4 (L)	Mi	Mo	Mi	0	0	0	Mo	Mi	0	0	0	0
5 (R)	0	0	0	0	0	0	Mo	0	0	0	0	0
5 (L)	Mi	Mi	0	0	0	0	Mo	Mi	Mi	0	0	0
**Discharge**												
**G1 (2 hours)**							G2 (6 hours)					
Days	D1	D2	D3	D4	D5	D6	D1	D2	D3	D4	D5	D6
1 (R)	0	0	0	0	0	0	S	0	0	0	0	0
1 (L)	Mi	0	0	0	0	0	S	Mo	Mi	0	0	0
2 (R)	Mi	0	0	0	0	0	0	0	0	0	0	0
2 (L)	S	Mo	0	0	0	0	S	0	Mi	0	0	0
3 (R)	Mo	Mo	0	0	0	0	Mi	Mo	0	0	0	0
3 (L)	Mi	Mo	0	0	0	0	Mo	Mi	Mi	0	0	0
4 (R)	Mo	Mo	Mi	0	0	0	S	Mo	Mi	0	0	0
4 (L)	0	Mo	Mi	0	0	0	Mi	Mi	0	0	0	0
5 (R)	0	0	0	0	0	0	S	Mo	0	0	0	0
5 (L)	0	0	0	0	0	0	S	S	Mo	Mi	0	0
**Photophobia**												
**G1 (2 hours)**							G2 (6 hours)					
Days	D1	D2	D3	D4	D5	D6	D1	D2	D3	D4	D5	D6
1 (R)	0	0	0	0	0	0	0	0	0	0	Mi	Mi
1 (L)	Mi	0	0	0	0	0	Mo	Mo	0	0	0	0
2 (R)	Mi	Mi	0	Mi	0	0	0	0	0	0	0	0
2 (L)	0	Mo	Mi	Mi	Mi	0	0	0	Mi	0	0	0
3 (R)	0	0	0	0	0	0	Mo	Mo	Mi	0	0	0
3 (L)	Mo	Mo	0	Mi	Mi	0	0	0	Mi	Mi	0	0
4 (R)	Mo	Mo	Mi	Mi	0	0	S	Mo	Mi	0	0	0
4 (L)	Mi	Mo	Mi	Mi	0	0	S	Mo	Mi	Mi	0	0
5 (R)	Mi	0	0	0	0	0	S	0	0	0	0	0
5 (L)	Mi	Mi	Mi	Mi	0	0	S	0	Mi	0	Mi	0


R; Right eye as test group, L; Left eye as control group, Mi; Mild, Mo; Moderate, and S; Severe.

The mean degree of conjunctival injection reduced by approximately 5-fold in both AMEED groups compared with the control group (P=0.05, Fig .2I). The mean discharge score in both AMEED groups was greater than the control group (by 3.77- and 1.17-fold, respectively), but the differences were not significant (P=0.05, Table 2). The mean degree of photophobia was also different in the AMEED groups compared with the control group. However, the differences were not significant (Table 2).

### Concentrations of growth factors in amniotic membrane extract eye drops

**Fig.3:**
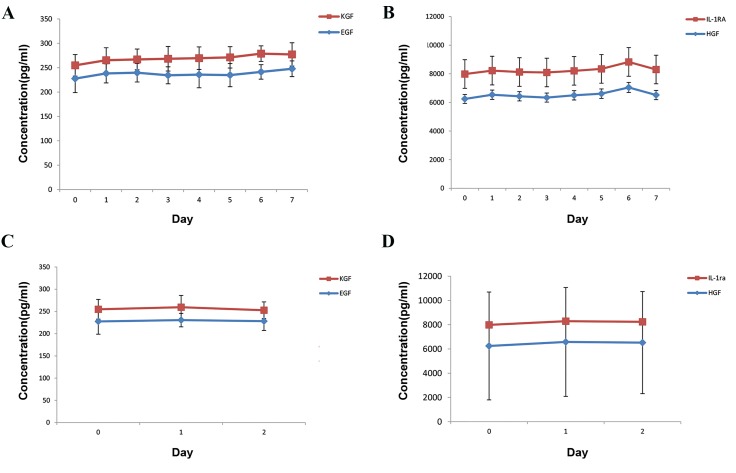
Stability of amniotic membrane extract eye drops (AMEED) during different time and temperature preservations. A, B. The graphs show stability of the AMEED growth factors for 7 days at 4°C (refrigerator) and 2 days, and C, D. At 25°C (room temperature).

The standardized AMEED could be used to promote corneal healing in patients or as a supplement in an ex vivo LSC culture. We analysed the concentration of growth factors (EGF, KGF, HGF, and IL-1RA) in four random batches of AMEED during different time points of preservation by using ELISA kits as previously described. However, we could not assess their bioactivities. The concentration of growth factors was stable during 7 days of storage at 2-8°C (refrigerator) and for a maximum of 2 days at 25°C (room temperature, P>0.05, Fig .3). Although the concentrations of growth factors varied by the time when preserved at -70, these differences were not significant for EGF (at least until 3 months), HGF and KGF (approximately 10 months), and IL-1RA (12 months) as seen in Table 3. In order to obtain a more complete understanding, the bioactivity of each growth factor should be evaluated under different conditions and time points.

**Table 3:** Concentration of growth factors in amniotic membrane extract eye drops (AMEED) pre- and post-storage at -70˚


	Pre-storage	Post-storage at -70˚C (pg/ml)			
		1.5months	3.0months	10months	12months

Epithelial growth factor (EGF)	211.15 ± 40.4	218.08 ± 53.8	186.73 ± 68.3	ND	ND
Hepatocyte growth factor (HGF)	3571.00 ± 1011.2	3511.00 ± 983.4	ND	2636 ± 1034.5	ND
Keratinocyte growth factor (KGF)	20.75 ± 22.2	ND	39.50 ± 13.8	44.50 ± 24.4	ND
Interleukin-1 receptor antagonist (IL-1RA)	1765.00 ± 195.4	1743.33 ± 156.7	1992.222	1360	1688.88


All data are mean ± SD (pg/ml). ND; Not determined.

## Discussion

The ideal method for LSC expansion and transplantation into patients who have CED should involve: i. A high level of safety with respect to the prevention of disease transmission, ii. Maintenance of LSC self-renewal capability; and iii. Ability of the LSCs to differentiate into corneal epithelial cells in a targeted manner (i.e., after transplantation) in order to protect the ocular surface (1719). FBS/foetal calf serum has been used in most previous clinical protocols for *ex vivo* human LSC expansion (20, 21). However, the use of FBS increases the risk of disease transmission and leads to unnecessary intracellular accumulation of bovine antigens (22) that can cause transplantation failure by inducing an immune response against the bovine antigens by the proliferating cells (23). Therefore, the use of autologous or cord blood serum (24, 25) and xeno- and serum-free culture conditions (26, 27) have been suggested.

The literature clearly indicates that HAMs in intact or extract forms are suitable to promote ocular surface reconstruction (28-31). However, few studies have explored the effectiveness HAM extracts on LSC cultures. Therefore, we have produced AMEED for use as a supplement for *ex vivo* LSC expansion. The supplement is free from animal products and other exogenous growth factors, which makes it an ideal candidate for enhancing *in vivo* LSC proliferation and for use as a topical treatment to heal corneal defects. We have tested the efficacy of AMEED for LSC expansion *ex vivo* and as treatment of corneal defects in an animal model.

Our results concurred with those by Dudok et al. (32) in 2015, which mentioned that human LSCs proliferated in tissue culture without the support of HAM. The *ex vivo* analysis revealed that the optimum dose of AMEED for LSC culture was 0.1 mg/ml and *in vivo* analysis indicated that the optimum dose in the rabbit model was 1 mg/ml. 

At these concentrations in the *ex vivo* culture, the expression of *ABCG2* significantly upregulated and the expression of *K3* significantly reduced. We showed that concentrations ≥2 mg/ml were cytotoxic; however, the lower concentration did not have any effect on proliferation. In a pilot study, we found that continuous addition of AMEED to culture medium led to a decrease in proliferation and an increase in apoptosis (data not shown). We found that AMEED appeared to limit LSC differentiation; however, this should be confirmed at the protein level by Western blot or flow cytometry analysis. 

These data indicated that the growth factor content of AMEED has a dose-dependent effect during culture and treatment; thus, accumulation of growth factors must be avoided. We assessed and verified the stability of the most important growth factors-EGF, KGF, HGF, and IL-1RA in AMEED under different conditions. The results showed that the growth factors in AMEED were stable for at least 10 months at -70°C, 7 days in a ref (pg/ml) rigerator (28 °C), and 2 days at room temperature. 

Subsequently, we tested the efficacy of 1 mg/ml AMEED to heal corneal defects in a rabbit model. The results showed that administration of AMEED every 2 hours was more effective than administration at 6-hour intervals. Previous research using *in vitro* wound-healing models has shown a positive dose-dependent effect of HAM suspensions on corneal re-epithelialization (10, 13, 33).

We believe that the cellular factors that make HAM transplantation an effective technique for the management of ocular disorders are present in AMEED. Our previous study has revealed that lumican, osteoglycin/memican, collagen a type IV, and fibrinogen were among the most abundant proteins in AMEED as well as lower concentrations of periplakin, pidogen 2, transglutaminase 2, and tubulointerstitial nephritis (2). Other researchers have reported that HAM contains EGF, KGF, HGF, and transforming growth factor (TGF) α and ß (34). These factors promote epithelial cell migration and differentiation, adherence between epithelial cells, and corneal epithelialization between the epithelium and basement membrane. It has been shown that AMEED leads to a significant increase in limbal epithelial cell migration and proliferation. It appears that AMEED can downregulate the activity of collagenoblasts (related to the effects of TGF-ß1, ß2-and, ß3) in wounds, prevent fibroblasts from converting to collagenoblasts, promote the restoration of the cornea propria, and decrease scarring (35-37). 

The preparation of AMEED is technically demanding (38); however, once prepared, AMEED can be stored for at least one year at -70°C. Therefore, in comparison to HAM transplantation, the use of AMEED is simpler, more convenient, and less likely to be associated with complications such as progression of the corneal surface disorder, calcification, inflammation and corneal thinning or perforation (39, 40). HAMs lyse 1-2 weeks after transplantation, thereby necessitating repeated transplantations. This issue is not observed with AMEED.

## Conclusion

AMEED increases LSC proliferation *ex vivo* and accelerates corneal epithelium healing *in vivo* without any adverse effects. Therefore, it could be used for corneal defect healing in humans and as a supplement in limbal or corneal cell therapy. 
